# Comparison of angle-to-angle distance and corneal diameter in pediatric eyes using ultrasound biomicroscopy

**DOI:** 10.1371/journal.pone.0305624

**Published:** 2024-06-18

**Authors:** Taylor D. Kolosky, Anusha U. Saga, Donald F. Dariano, Urjita Das, Bhakti K. Panchal, Jana A. Bregman, Moran R. Levin, Janet Leath Alexander

**Affiliations:** 1 Department of Ophthalmology and Visual Sciences, University of Maryland School of Medicine, Baltimore, Maryland, United States of America; 2 Department of Medicine, Temple University Hospital, Philadelphia, Pennsylvania, United States of America; 3 Department of Biochemistry and Molecular Medicine, West Virginia University School of Medicine, Morgantown, West Virginia, United States of America; Alexandria University Faculty of Medicine, EGYPT

## Abstract

**Objective:**

To investigate the relationship between corneal diameter and internal corneal span determined from angle-to-angle distance using ultrasound biomicroscopy (UBM) in an observational cross-sectional patient population comprised of 54 eyes (28 healthy control eyes, ages 0.1 to 11.3 years; 26 eyes with primary congenital glaucoma, ages 0.1 to 3.5 years) from 41 pediatric participants ages 0.1 to 11.3 years (mean age: 3±3 years, median age: 2 years).

**Methods:**

Forty cornea photographs with reference ruler and 110 UBM images were obtained. Three observers measured horizontal and vertical corneal diameter and angle-to-angle distance in each cornea photo and UBM image using ImageJ and the average values were used. Main outcome measures were Pearson correlation coefficient, linear regression, mean difference between corneal diameter and angle-to-angle distance, and intra-class correlation coefficients among measurements from all three observers for each parameter.

**Results:**

Corneal diameter and angle-to-angle distance had a strong positive correlation horizontally (Pearson r = 0.89, p<0.001) and vertically (r = 0.93, p<0.001). Correlation was consistent regardless of presence of primary congenital glaucoma and participant age. Regression analysis demonstrated a linear relationship between the parameters for horizontal (CD = 0.99*AA+0.28, R2 = 0.81, p<0.001) and vertical (CD = 0.91 *AA+1.32, R2 = 0.85, p<0.001) dimensions. Overall, reliability was good-excellent, ranging from an ICC of 0.76 for vertical corneal diameter to 0.90 for horizontal angle-to-angle distance.

**Conclusions:**

Based on the strong positive correlation found between corneal diameter and angle-to-angle distance in our study population, UBM image analysis can be used to accurately estimate corneal diameter from angle-to-angle distance in children with healthy eyes and primary congenital glaucoma. UBM may provide a useful intraocular alternative for estimating corneal diameter and monitoring diseases that affect the cornea in infants and children, such as congenital glaucoma.

## Introduction

Quantitative corneal diameter evaluation is pertinent to the diagnosis and monitoring of several ophthalmic diseases presenting in pediatric patients, such as congenital glaucoma, microcornea, microphthalmos, and megalocornea [1]. Increased corneal diameter is often one of the first indicators of elevated intraocular pressure (IOP) in infants and young children, which can aid in the timely diagnosis and assessment of congenital glaucoma [2–4]. Corneal diameter is also an essential biometric parameter during cataract surgery planning, as intraocular lens (IOL) size may be selected based on this measurement [5]. Incorrect sizing can result in complications such as uveitis, secondary glaucoma, and endothelial injury [5]. Thus, accurate measurement of corneal diameter is imperative.

Anterior segment measurements, including corneal diameter, are commonly obtained using automated devices such as the IOLMaster (Carl Zeiss, Meditec, AG) and the Orbscan IIz (Bausch & Lomb). While evaluating younger patients for whom this approach may not be feasible, corneal diameter is typically measured during examination under anesthesia (EUA) using calipers or a ruler. Although widely accepted as the current standard, this method of measurement is subject to error and inter-observer variability [6]. Prior studies have examined the correlation between anterior segment imaging modalities and external corneal diameter, but findings have been inconclusive and often contradictory [5, 7, 8]. However, these studies primarily focused on adult subjects and used techniques such as anterior segment optical coherence tomography (AS-OCT). Other instruments, such as ultrasound biomicroscopy (UBM), have yet to be explored for this purpose.

Ultrasound biomicroscopy (UBM), a high-frequency sonographic technique, has been established as a clinically valuable imaging modality for structural anterior and posterior segment assessment, with the unique capability for intraocular visualization even in cases of corneal clouding or opacity [9]. UBM has significant utility in the pediatric population, given that children often have anomalies posterior to the iris or anterior segment disease accompanied by corneal haze [10]. In addition, UBM image analysis can be used to extract clinically meaningful ocular measurements with high reliability, such as central corneal thickness and anterior chamber depth, that can guide diagnosis and treatment decisions [11]. This niche renders UBM particularly promising regarding its potential to monitor congenital disease progression in children using intraocular measurements. Compared to traditional measurement techniques for corneal diameter, UBM allows for a more comprehensive observation of the eye with high reliability in intraocular measurements; therefore, UBM may be a useful, more versatile alternative that provides more information about the eye that can inform medical decision-making.

This prospective, cross-sectional study was conducted to investigate the relationship between corneal diameter (CD) and internal corneal span in pediatric patients determined from the angle-to-angle distance (AA) measured in UBM images.

## Materials and methods

### Participants

This study adhered to the ethical principles outlined in the Declaration of Helsinki as amended in 2013. The University of Maryland, Baltimore (UMB) Institutional Review Board (IRB) has approved this study protocol and the corresponding consent forms. Written informed consent for participation in this study was obtained from the parent or guardian of each prospective participant. Collection and evaluation of protected health information was compliant with the Health Insurance Portability and Accountability Act of 1996.

Fifty-four eyes from 41 pediatric participants aged 0.1 to 11.3 years (mean age: 3±3 years, median age: 2 years) were included in this study. Twenty-eight subjects ages 0.1 to 11.3 years contributed 28 healthy eyes and 13 subjects ages 0.1 to 3.5 years with primary congenital glaucoma contributed 26 eyes. None of the subjects with glaucoma had undergone surgery at the time of imaging. Seven of the subjects (13 eyes) were being treated with one or more medications for glaucoma, including acetazolamide, dorzolamide/timolol, latanoprost, and pilocarpine. Our cohort was comprised of prospective subjects who underwent UBM imaging at our institution between November 1st, 2014, and May 31st, 2022. Participants were consented and enrolled during this period prior to imaging.

Congenital glaucoma was recognized as glaucoma diagnosis prior to the age of two years. The diagnosis of glaucoma required one or more of the following structural changes: (1) increased corneal diameter (greater than 11.0 mm before age 1 year or greater than 12.0 mm between age 1–2 years), or greater than 1.5 mm of asymmetry of corneal diameter, (2) progressive myopic shift concurrent with an increase in corneal diameter and/or axial length, (3) increased optic nerve cupping by 20% or greater (as measured using cup-to-disc ratio), or (4) the need for surgical intervention for IOP control. Subjects with a history of anterior segment trauma were excluded.

Healthy controls were subjects without a history of glaucoma presenting for care of a contralateral eye or eyelid condition, or ophthalmic indications unrelated to the anterior segment. No control subjects had any history of intraocular surgery or traumatic injury to the enrolled eye; however, controls presented with contralateral conditions such as trauma, strabismus, ptosis, nasolacrimal duct obstruction, or orbital cyst. All control subjects demonstrated age-appropriate visual acuity and visual behavior, normal comprehensive eye examination, and normal dilated fundus exam by a board-certified pediatric ophthalmologist.

### Imaging and image analysis

All subjects were imaged under general anesthesia prior to planned surgical procedure. The Alfonso eyelid speculum was used for subjects imaged under general anesthesia. Cotton-tipped applicators were used for globe positioning if needed. Prior to imaging subjects, the operator captured a digital photo of the eye that allowed clear visualization of the entire corneal limbus. A reference ruler was also included in each photograph. Ultrasound biomicroscopy imaging was performed by an experienced operator, a trained pediatric ophthalmologist, using the Aviso Ultrasound Platform A/B ultrasound biomicroscopy with a 50-MHz linear transducer (Quantel Medical, Clermont-Ferrand, France). For all subjects, the transducer was covered with a single-use ClearScan UBM probe cover filled with deionized water. Hypromellose ophthalmic solution (2.5%) was applied to the ocular surface as lubrication. No subject received pharmacologic dilation before imaging. The operator collected at least one horizontal axial and at least one vertical axial ultrasound image per eye, concurrent with predetermined landmarks and probe positions from an established protocol [11]. This included performing a sweep of the anterior chamber to aid in determining the dimensions with the highest anterior chamber depth.

The manual image analysis protocol uses ImageJ 1.48v (National Institutes of Health), a Java-based open access image processing program. Reliability and repeatability analysis of the current imaging and analysis protocol has been previously published [11]. Images were de-identified and reviewed for eligibility. Corneal photos that did not clearly capture the entire limbus were excluded. Ultrasound images were selected based on centration of the pupil, ability to identify the angles, and overall quality. Three independent trained observers measured each corneal photo and UBM image, and the average measurements were used for analysis.

**Fig 1:**
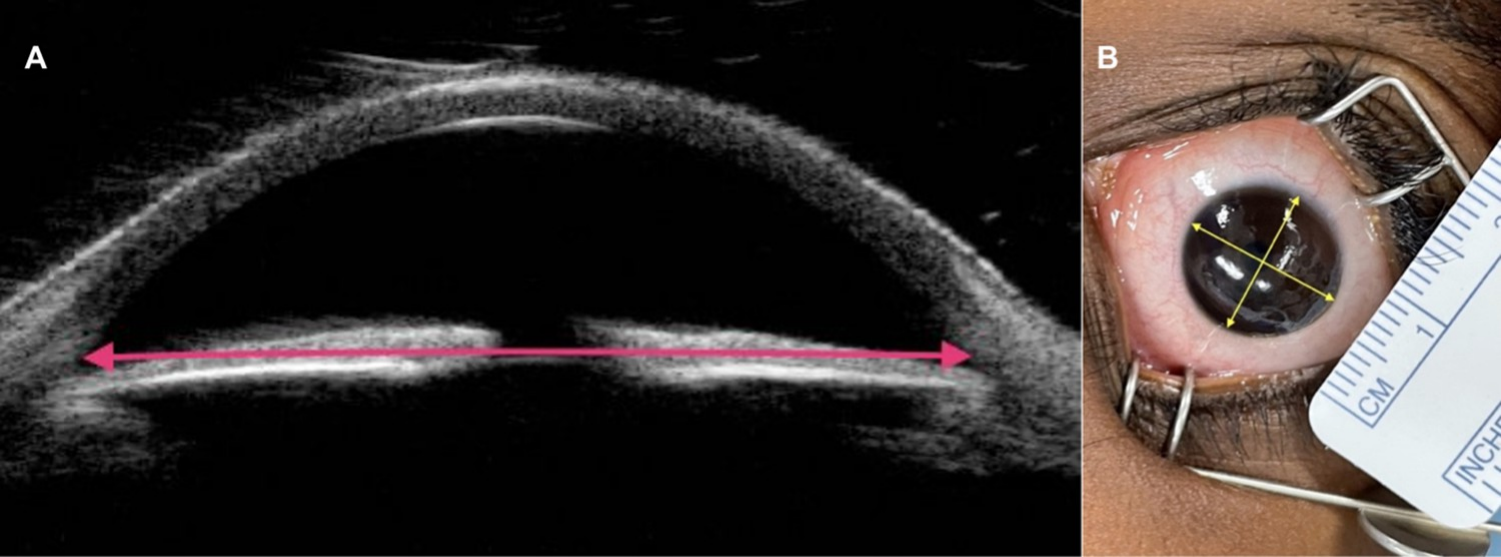
Angle-to-angle and corneal diameter measurement techniques. **(A)** Angle-to-angle (AA) distance measured in a vertical axial UBM image; **(B)** Horizontal and vertical corneal diameter (CD) measured in a cornea photograph of a six-year-old child without ocular disease.

Measured parameters included angle-to-angle distance in each UBM image, defined as the diameter between the iridocorneal angle’s opposing recesses (Fig 1A), and horizontal and vertical corneal diameters, or white-to-white distance, in each photograph (Fig 1B). For subjects that did not have corneal photos with reference ruler available (n = 19 eyes), calipers verified by ruler were used to measure corneal diameter. Previous studies have found acceptable levels of agreement between these two methods of corneal diameter measurement [6, 12].

### Statistical analysis

The mean, median, and standard deviation of the horizontal CD, vertical CD, horizontal AA, and vertical AA were calculated for the study population, the sample of healthy control subjects, and the sample of subjects with glaucoma. The 95% confidence intervals (CI) of the mean were calculated for each parameter. Normality testing was performed on the healthy control, glaucoma, and overall datasets using joint normality calculated through Henze-Zirkler testing prior to statistical analysis.

Linear regression analysis was performed between horizontal CD and AA and vertical CD and AA for the study population, the control group, and the glaucoma group. Subjects were further divided based on their age and regression analysis was performed for each sub-group. The age groups were defined as follows: <1 y.o. (N = 11), 1–2 y.o. (N = 8), 2–3 y.o. (N = 6), 3–4 y.o. (N = 5), and 5–11 y.o. (N = 11). Analysis of variance (ANOVA) was conducted to determine whether the differences in regression lines across age groups were statistically significant. Statistical significance was set at a p-value of 0.05.

Pearson correlation coefficient was calculated for horizontal CD and AA, and vertical CD and AA for the study population, the control group, and the glaucoma group. One-way mixed effects inter-class correlation coefficients (ICC) were calculated for each parameter to assess agreement and reliability of measurements among all three observers. The Bland-Altman method was used to evaluate the agreement between CD measurements from photographs and AA measurements from UBM images [13]. R Studio Version 2023.06.0+421 was used for all statistical analyses.

## Results

Forty digital external photographs (33 healthy control eye photos, 7 glaucoma eye photos) and 110 ultrasound biomicroscopy images (56 healthy control eye images, 52 glaucoma eye images) from 54 eyes of 41 pediatric participants were included in the analysis. Subject demographics are summarized in Table 1.

**Table 1:** Participant demographics.

	All participants (N = 41)	Control (N = 28)	Glaucoma (N = 13)
**Eyes,** n	54	28	26
**Female sex,** N (%)	24 (58.5)	18 (64.3)	6 (46.2)
**Mean age, y** (SD)	3.2 (3.1)	4.2 (3.2)	1.0 (1.2)
**Median age, y**	2.2	3.1	0.3
**Age range, y**	0.1–11.3	0.3–11.3	0.1–3.5
**Black race,** N (%)	22 (53.7)	14 (50.0)	9 (69.2)
**White race,** N (%)	12 (29.3)	10 (35.7)	2 (15.4)
**Race not reported**	3 (7.3)	2 (7.1)	1 (7.7)
**Hispanic/Latino,** N (%)	5 (12.2)	3 (10.7)	2 (15.4)

Demographics of all study participants.

n = number of eyes

N = number of participants

SD = standard deviation

Mean, median, and range of measurements for horizontal and vertical CD and AA are shown in Table 2.

**Table 2:** Descriptive summary.

	All participants (N = 41)	Control (N = 28)	Glaucoma (N = 13)
**Mean horizontal CD, mm (SD) [95% CI]**	11.8 (0.9) [10.1,13.5]	11.5 (0.6) [9.9, 13.7]	12.2 (1.1) [10.1, 14.4]
**Median horizontal CD, mm**	11.7	11.5	12.3
**Horizontal CD range, mm**	10.1–14.0	10.1–12.8	10.5–14.0
**Mean vertical CD, mm (SD) [95% CI]**	11.6 (0.8) [10.2, 13.0]	11.3 (0.4) [10.1, 13.1]	12.0 (0.9) [10.1, 13.8]
**Median vertical CD, mm**	11.4	11.3	11.9
**Vertical CD range, mm**	10.5–13.5	10.5–12.1	10.6–13.5
**Mean horizontal AA, mm (SD) [95% CI]**	11.6 (0.8) [10.1, 13.1]	11.4 (0.6) [10.0, 13.3]	11.9 (1.0) [9.8, 13.9]
**Median horizontal AA, mm**	11.6	11.6	12.0
**Horizontal AA range, mm**	10.0–13.9	10.0–12.8	10.0–13.9
**Mean vertical AA, mm (SD) [95% CI]**	11.6 (0.7) [9.9, 12.8]	11.3 (0.4) [10.2, 12.9]	11.69 (0.85) [10.2, 13.5]
**Median vertical AA, mm**	11.4	11.3	11.46
**Vertical AA range, mm**	10.5–13.8	10.6–12.5	10.38–13.46

Summary of corneal diameter and angle-to-angle measurements in all participants.

N = number of participants

SD = standard deviation

CI = confidence interval

Overall, reliability among raters ranged from good (ICC = 0.76, vertical CD) to excellent (ICC = 0.90, horizontal AA). The ICC values for all parameters are reported in Table 3.

**Table 3:** Inter-observer agreement.

	ICC [95% CI]
**Horizontal CD**	0.804 [0.697, 0.91]
**Vertical CD**	0.761 [0.675, 0.85]
**Horizontal AA**	0.895 [0.791, 0.951]
**Vertical AA**	0.787 [0.699, 0.847]

Inter-class correlation coefficients for corneal diameter and angle-to-angle measurements.

ICC = inter-class correlation coefficient

CI = confidence interval

**Fig 2:**
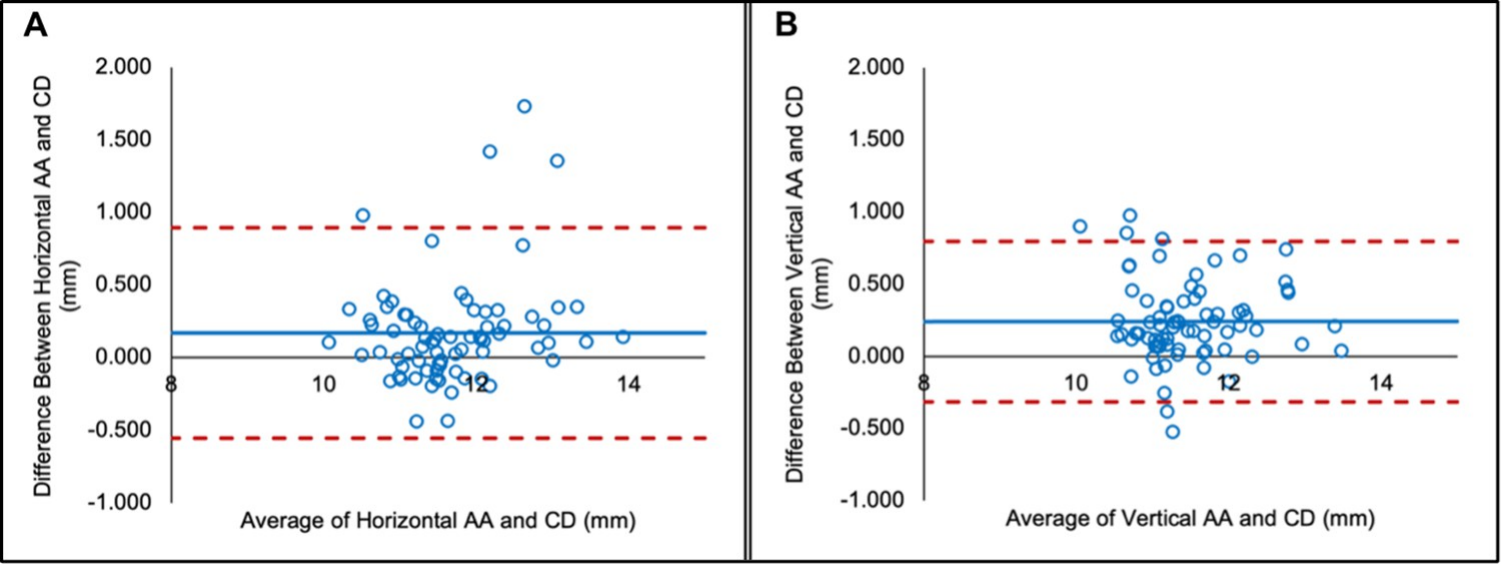
Agreement between angle-to-angle and corneal diameter measurements. Bland-Altman plots depicting the agreement between **(A)** horizontal corneal diameter (CD) and horizontal angle-to-angle (AA) measurements; **(B)** vertical CD and vertical AA measurements. The solid line represents the mean difference (bias), and the dotted lines represent 95% LoA.

Bland-Altman analysis demonstrated high agreement between CD and AA for horizontal and vertical measurements in all 54 eyes (Fig 2), with mean differences of 0.14 and 0.06 mm, respectively. The 95% limits of agreement (LoA) for horizontal and vertical dimensions were −0.41 to 0.69 mm and -0.49 to 0.61 mm.

Horizontal CD and AA were strongly positively correlated in all 54 eyes (Pearson r = 0.89), in the healthy control subgroup (r = 0.95), and the glaucoma subgroup (r = 0.88). Correlation analysis also demonstrated strong positive correlation between vertical CD and AA in all 54 eyes (r = 0.93), the control subgroup (r = 0.91), and the glaucoma subgroup (r = 0.92).

**Fig 3:**
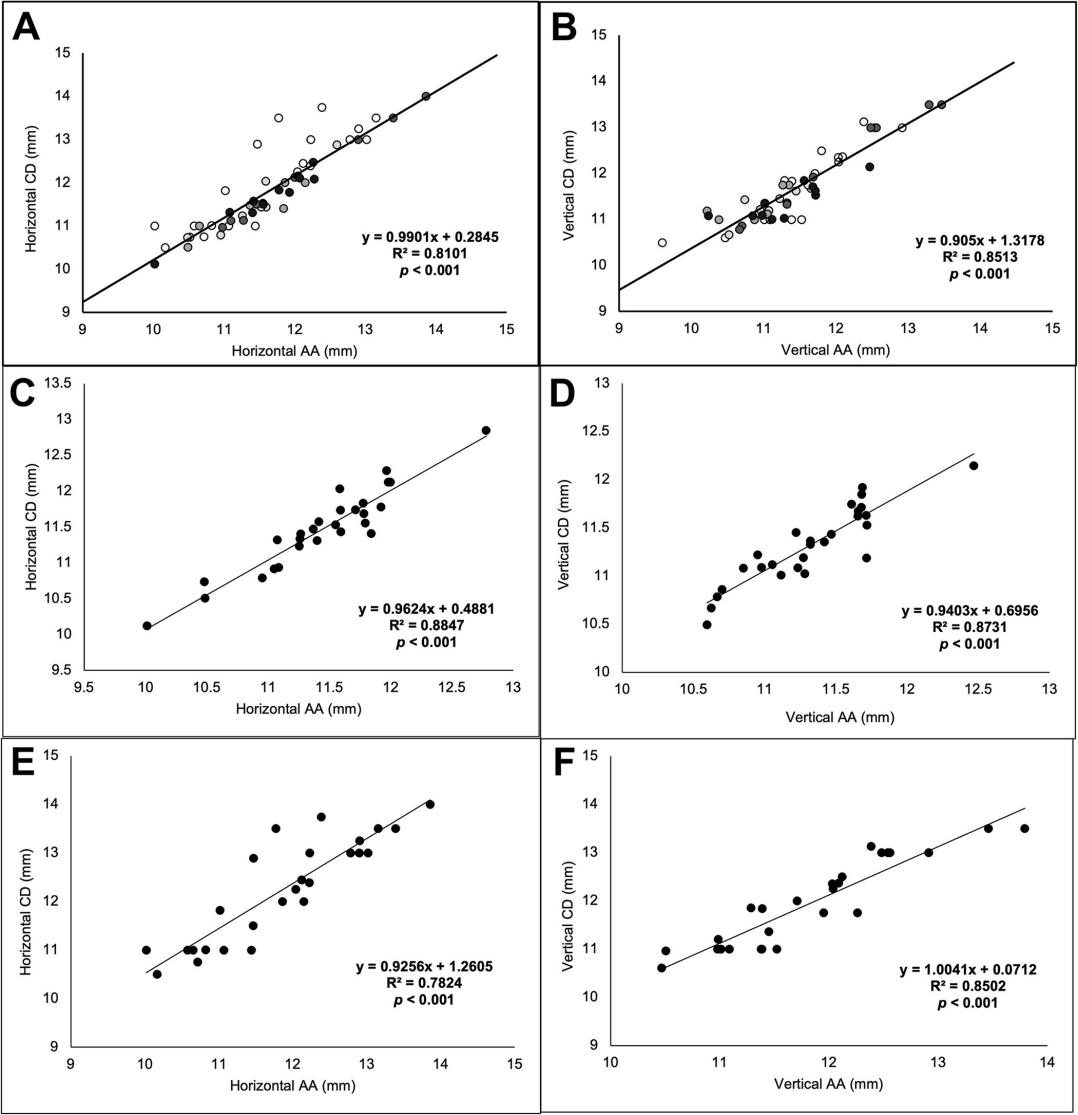
Linear relationship between angle-to-angle and corneal diameter. Regression plots depicting the relationship between corneal diameter (CD) and angle-to-angle (AA) measurements for: **(A)** horizontal and **(B)** vertical measurements for all eyes, where subject age is represented by shade, with darker shades corresponding with older children; **(C)** horizontal and **(D)** vertical measurements for healthy control subjects; **(E)** horizontal and **(F)** vertical measurements for subjects with glaucoma.

Regression analysis including all 54 eyes showed a linear relationship between AA and CD for horizontal and vertical measurements that was robust to subject age (Fig 3A and 3B). Analysis of variances (ANOVA) demonstrated no significant difference in linearity among age groups. Linearity between AA and CD was observed in both the healthy control (Fig 3C and 3D) and glaucoma sub-groups (Fig 3E and 3F).

Correlation coefficients (*R* values) and regression equations are displayed in Table 4.

**Table 4:** Pearson correlation and linear regression.

	All participants (N = 41)	Control (N = 28)	Glaucoma (N = 13)
**Horizontal CD vs. AA Pearson r** *	0.89	0.95	0.88
**Horizontal CD vs. AA regression equation (R** ^**2**^ **)**	CD = 0.99*AA + 0.28 (0.81)	CD = 0.96*AA + 0.49 (0.88)	CD = 0.93*AA + 1.26 (0.78)
**Vertical CD vs. AA Pearson r** *	0.93	0.91	0.92
**Vertical CD vs. AA regression equation (R** ^**2**^ **)**	CD = 0.91*AA + 1.32 (0.85)	CD = 0.94*AA + 0.70 (0.87)	CD = 1.00*AA + 0.07 (0.85)

Pearson correlation coefficients and regression equations for corneal diameter and angle-to-angle relationship.

*p-value of 0.05 used

N = number of subjects

## Discussion

The purpose of this study was to examine the correlation between corneal diameter and interior corneal span, or angle-to-angle distance, in 41 pediatric subjects using external photographs and ultrasound biomicroscopy. We hypothesized that AA would be strongly correlated with CD, and that this relationship would allow conversion between internal (AA) and external (CD) measures of corneal span. We studied this relationship in a cohort of 28 pediatric subjects with healthy eyes and a cohort of 13 subjects with congenital glaucoma and demonstrated a strong positive correlation between the two ocular parameters that was robust to age. Not only will these findings improve understanding of the association between intraocular dimensions and corneal characteristics, but they will also highlight the clinical utility of UBM in the diagnosis and monitoring of congenital diseases that affect the cornea, such as pediatric glaucoma.

Measurements obtained for corneal diameter and angle-to-angle distance were mostly in agreement with published anatomical values for pediatric patients with healthy eyes and pediatric patients with congenital glaucoma, although glaucoma patients tended to have smaller corneas than reported in the literature for their population [2, 14–17]. Our results agree with prior studies which demonstrated horizontal measures of corneal diameter and angle-to-angle distance tending to be greater than corresponding vertical measures [18, 19].

Our investigation has demonstrated angle-to-angle distance measured from UBM images to be a strong predictor of corneal diameter in pediatric subjects with healthy eyes and glaucoma. Horizontal and vertical corneal diameter and angle-to-angle measurements were highly positively correlated in the healthy controls and subjects with glaucoma. We demonstrated a slight tendency toward greater corneal diameter measurements than angle-to-angle measurements, which was expected due to the thickness of the peripheral cornea and is consistent with published normative data [18, 20, 21]. Regression analysis demonstrated strong positive linear relationships between horizontal corneal diameter and angle-to-angle, and vertical corneal diameter and angle-to-angle, in all 54 eyes. This relationship remained consistent after separating subjects by age group and diagnosis. However, given that there were few subjects available to comprise certain age groups, such as children aged 3–4 years and 4–5 years, it is difficult to draw conclusions about those age groups from this data.

To our knowledge, this is the first study that uses UBM to examine the correlation between corneal diameter and angle-to-angle distance [22, 23]. Several studies have compared these dimensions in adult participants using OCT, with variable results. Piñero et al. found a statistically significant, although weak, positive correlation between horizontal corneal diameter and angle-to-angle distance using a digital caliper and the Visante OCT in 30 healthy eyes of 19 adults [5]. However, the authors reported significant variability within their relatively small dataset, preventing extrapolation. In a study by Kohnen et al., there was a strong linear relationship between horizontal angle-to-angle distance and corneal diameter, measured using the Visante OCT and a combination of the Orbscan IIz (Bausch & Lomb) and IOLMaster (Carl Zeiss Meditec) [7]. Using the IOLMaster and AS-OCT, Nemeth et al. found horizontal corneal diameter and angle-to-angle to be positively correlated [21].

In contrast, Goldsmith et al. only found a weak correlation between horizontal angle-to-angle distance and corneal diameter in 20 adults, although researchers used an experimental CAS OCT system and a Holladay-Godwin cornea gauge instead of manual image analysis [8]. Notably, Kawamorita et al. [22] reported poor agreement between corneal diameter and horizontal ciliary sulcus diameter measured using UBM in a group of 31 healthy adults; however, they used scanning-slit topography to obtain corneal diameter, which has yielded variable measurements compared to other techniques, such as calipers or the IOLMaster [6, 24]. Limited data exists on the relationship between these parameters along the vertical meridian, most likely related to difficulties in obtaining vertical corneal diameter measurements with standard techniques due to the eyelids obstructing the view of the limbus. However, we found longitudinal angle-to-angle distance and vertical corneal diameter to have a strong correlation.

Our findings are the most consistent with those of Kohnen and Nemeth [7, 21]. Notably, direct comparisons between prior studies and the present work should be made with caution, considering the variety of different methods used to measure corneal diameter and angle-to-angle distance. In addition, many previous studies did not utilize comparable subject populations (i.e., most studies used adult participants and included less than 54 eyes).

There were limitations inherent to this paper. Our study includes a relatively modest patient population. We were limited by few healthy control subjects under one year old undergoing general anesthesia. Similarly, due to the limited population, our study does not provide demographic-specific data, despite established ethnicity differences in corneal and anterior chamber parameters in pediatric patients [25–29]. Ethnicity-based data would be a valuable addition to future studies examining the relationship between angle-to-angle distance and corneal diameter in children.

In addition, a manual measurement method, which is subject to intra- and inter-operator variability, was used to determine corneal diameter. Despite relying upon manual measurements, our inter-observer reliability for corneal diameter and angle-to-angle measurements was good-excellent, which may be attributed to consistent and thorough observer training. However, intrasession repeatability analysis should have been considered and included in this prospective study. Future comparative studies could use automated grey-scale devices such as the IOLMaster and the Orbscan IIz, which have produced more repeatable measures of corneal diameter than caliper and ruler [6]. However, these studies would be limited to older children who would be able to cooperate with an exam involving these modalities. Furthermore, measurements of the vertical meridian would be more difficult to obtain due to limitations associated with these techniques.

Future studies should aim to examine the relationship between longitudinal angle-to-angle distance and progression of congenital glaucoma, a disease known to impact corneal diameter. Given the strong correlation demonstrated between angle-to-angle distance and corneal diameter in this study, we would expect increased angle-to-angle distance to be an indicator for progression of congenital glaucoma. In addition, the linear anatomical relationship defined in our paper should be applied to a new dataset to validate these findings.

In conclusion, ultrasound biomicroscopy can be used to accurately estimate corneal diameter in pediatric eyes, as shown by the strong correlation and linear relationship between corneal diameter and angle-to-angle measurements in both cohorts. This association is robust to age, with no significant differences in linearity from infancy to early adolescence in our sample. Given its high resolution and versatile nature, ultrasound biomicroscopy may be a useful alternative for estimating corneal diameter from longitudinal angle-to-angle distance. These results should be interpreted in the context of the study’s main limitations; our patient sample was relatively small and may not have been fully representative of the congenital glaucoma patient population. Further studies with more eyes and more patients with congenital glaucoma are needed to confirm these findings, which partially contradict results in the current literature.

## Supporting information

S1 File Dataset. Dataset containing cornea diameter and angle-to-angle measurements from all eyes.
(CSV)
